# Yellow fever in a traveller returning from Suriname to the Netherlands, March 2017

**DOI:** 10.2807/1560-7917.ES.2017.22.11.30488

**Published:** 2017-03-16

**Authors:** Marjan Wouthuyzen-Bakker, Marjolein Knoester, Aad P van den Berg, Corine H GeurtsvanKessel, Marion PG Koopmans, Coretta Van Leer-Buter, Bob Oude Velthuis, Suzan D Pas, Wilhelmina LM Ruijs, Jonas Schmidt-Chanasit, Stephen GS Vreden, Tjip S van der Werf, Chantal BEM Reusken, Wouter FW Bierman

**Affiliations:** 1 University of Groningen, University Medical Center Groningen, Department of Internal Medicine / Infectious Diseases, Groningen, The Netherlands; 2 These authors contributed equally to this work; 3 University of Groningen, University Medical Center Groningen, Department of Medical Microbiology and Infection Prevention, Groningen, The Netherlands; 4 University of Groningen, University Medical Center Groningen, Department of Gastroenterology & Hepatology, Groningen, The Netherlands; 5 Erasmus MC, Department of Viroscience, WHO Collaborating Centre for Arbovirus and Haemorrhagic Fever Reference and Research, Rotterdam, The Netherlands; 6 University of Groningen, University Medical Center Groningen, Department of Critical Care, Groningen, The Netherlands; 7 National Coordination Centre for Communicable Disease Control, Centre for Infectious Disease Control, National Institute for Public Health and the Environment (RIVM), Bilthoven, the Netherlands; 8 Bernhard Nocht Institute for Tropical Medicine, WHO Collaborating Centre for Arbovirus and Haemorrhagic Fever Reference and Research, Hamburg, Germany; 9 German Centre for Infection Research (DZIF), partner site Hamburg-Luebeck-Borstel, Hamburg, Germany; 10 Academic Hospital Paramaribo, Department of Internal Medicine and Infectious Diseases, Paramaribo, Suriname

**Keywords:** The Netherlands, Suriname, vector-borne infections, viral infections, yellow fever, yellow fever virus, travel, clinic, laboratory

## Abstract

A Dutch traveller returning from Suriname in early March 2017, presented with fever and severe acute liver injury. Yellow fever was diagnosed by (q)RT-PCR and sequencing. During hospital stay, the patient’s condition deteriorated and she developed hepatic encephalopathy requiring transfer to the intensive care. Although yellow fever has not been reported in the last four decades in Suriname, vaccination is recommended by the World Health Organization for visitors to this country.

Yellow fever virus (YFV) is known to be enzootic in South America, causing periodic outbreaks of disease in monkeys and humans in some countries. In Brazil, there has been an outbreak of yellow fever ongoing since December 2016 with 1,500 cases as at 9 March [1,2]. Here we report an imported case of human infection with YFV in a traveller returning from Suriname, on the north-eastern coast of South America, from where the last case of yellow fever was reported 45 years ago.

## Case description

In March 2017, a Dutch Caucasian female in her late 20s from the Netherlands was referred to the University Medical Center Groningen in the Netherlands because of high fever and signs of severe acute liver injury after returning from a two-week stay in Suriname. She had no co-morbidities apart from obesity (body mass index around 40 kg/m^2^, norm: 18.5–25 kg/m^2^). During her visit she stayed in the capital of Suriname, Paramaribo, and she made several daytrips by boat and car, of which two in the tropical rainforest (Figure).

**Figure:**
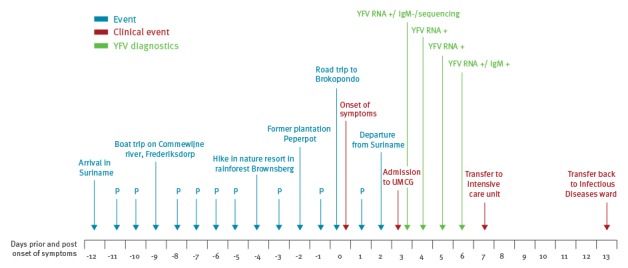
Timeline of events and diagnostic results, case of yellow fever in a traveller returning from Suriname to the Netherlands, March 2017

P: Paramaribo; RNA: ribonucleic acid; UMCG: University Medical Center Groningen; YFV: yellow fever virus.

She recalled having been bitten by mosquitoes during her hike at Brownsberg, a nature resort in the rainforest with wildlife. Before her travel, she did not visit a travel clinic and did not receive yellow fever vaccination. On day 12 of her visit in Suriname, she experienced mild muscle pain, headache and nausea and she developed a high-grade fever. She returned to the Netherlands on day 15 and visited the emergency department of a secondary care centre, from where she was referred to our University hospital. At physical examination she was not icteric. Except for a temperature of 39.9 °C, vital parameters were normal. The results of the remaining physical examination were unremarkable. Laboratory testing revealed leukopenia (leukocytes 0.9x10^9^/L, norm: 4.0–10.0x10^9^/L) and massive liver injury (aspartate aminotransferase 5,787 U/L, norm: <31 U/L; alanine aminotransferase 4,910 U/L, norm: <34 U/L), with mildly elevated bilirubin levels (total bilirubin 20 µmol/L, norm: <17 µmol/L). Liver synthesis was impaired as revealed by increased clotting times (activated partial thromboplastin time (APTT): 49s, norm: 23–33s; prothrombin time (PT): 26.6s, norm: 9.0–12.0s) and reduced antithrombin (49%, norm: 80–120%). Fibrinogen was diminished suggestive of diffuse intravascular coagulation. Renal function was normal apart from severe albuminuria (up to 22.6 g/24h, norm: 0g/24h). Malaria, viral hepatitis (A, B, C, E, Epstein Barr virus, cytomegalovirus, herpes simplex virus), dengue, chikungunya and Zika were ruled out (Table). Diagnostic tests to exclude leptospirosis performed on day 6 post onset of symptoms (dps 6) were inconclusive (Table) and a convalescent serum was going to be tested at the time of publication. Because of the combination of fever, leukopenia, thrombocytopenia, liver injury and travel history, yellow fever was included in the differential diagnosis. Real-time reverse transcriptase PCR (qRT-PCR) was positive for YFV in serum taken on dps 3. On dps 7 the patient’s condition deteriorated due to hepatic encephalopathy (ammonia 149 µmol/L, norm: 15–45 µmol/L). Cerebral oedema and bleeding was ruled out by computed tomography (CT)-scan. The patient was transferred to the intensive care unit for close observation of vital parameters. Vitamin K was administered. Hepatic encephalopathy was treated with rifaximin and lactulose. Ceftriaxone (2g per day intravenously) was given for 7 days as antibiotic prophylaxis. Consequently, possible leptospirosis was also treated. Her neurological condition stabilised on dps 10 together with the coagulation parameters. On dps 13 the patient was transferred back to the ward.

**Table:** Pathogens for which laboratory tests were performed, yellow fever case, the Netherlands, March 2017

Pathogen	Blood (day 3 post onset of symptoms)
*Plasmodium* spp.	Thick smear negative, antigen test negative
Hepatitis A virus	IgM and IgG negative
Hepatitis B virus	Serological screening negative
Hepatitis C virus	Serological screening negative
Hepatitis E virus	PCR negative
Epstein Barr virus	IgM and IgG negative
Cytomegalovirus	IgM and IgG negative
Herpes simplex virus type 1 and 2	PCR negative
Dengue virus	PCR negative, IgM and IgG negative
Chikungunya virus	PCR negative, IgM and IgG negative
Zika virus	PCR negative, IgM and IgG negative^a^
*Leptospira *spp.	PCR negative, microscopic agglutination test negative, IgM 1:80^b^

^a^ Performed on day 5 post onset of symptoms (dps 5).

^b^ ELISA (in-house ELISA Dutch Leptospirosis Reference Center) performed on dps 6 showed IgM 1:80 (cut-off positive IgM ≥1:160). IgM results were negative on dps 3 and dps 7 using Leptocheck-WB (Zephyr Biomedicals, Goa, India).

### Virology findings

qRT-PCR and/or pan-flavivirus RT-PCR on blood samples on dps 3 did not detect chikungunya virus (CHIKV), dengue virus (DENV), or Zika virus (ZIKV) (Table) [3,4]. In four consecutive samples of dps 3–6, YFV-RNA was detected (Figure) [4-6], with increasing Ct values (from 23 to 31 from dps 3 to dps 5 [5] and 39 on dps 6 [6]). Sequencing of a 176 bp pan-flavivirus hemi-nested RT-PCR product, targeting part of the NS5 genomic region confirmed YFV infection [4]. The sequence was deposited in the GenBank database under the following accession number: KY774973.

On dps 3, indirect immunofluorescence assays (IFA) was negative for IgM and IgG against YFV (Flavivirus Mosaic, Euroimmun AG, Luebeck, Germany). A convalescent sample of dps 6 was clearly positive for YFV IgM (titre 1:10, Figure), with non-reactive IgG. This anti-YFV IgM response on dps 6 is in line with literature stating that IgM antibodies usually appear during the first week of illness. Neutralising IgG antibodies are likely to appear towards the end of the first week after onset of illness and will be tested for in convalescent serum [7].

## Background

YFV is a mosquito-borne virus in the genus Flavivirus, family *Flaviviridae*, related to DENV, ZIKV, tick-borne encephalitis virus and West Nile virus. YFV is maintained in a sylvatic cycle between non-human primates and so-called ‘jungle’-mosquitoes (*Hemagogus* and *Sabethes* spp. in South America) [8]. Sporadic infection of humans with sylvatic YFV can occur when unprotected humans are exposed while entering the habitats where the viruses circulate. Subsequent introduction of a viraemic human case to urban areas with high population densities and *Aedes aegypti* mosquitoes can initiate an urban transmission cycle [9]. YFV is endemic in (sub)tropical areas of South America and Africa. The risk for YFV infection in South America is the highest in tropical regions and during the rainy season (January–May) when mosquito population densities peak [10]. In 2011, Suriname was identified by the World Health Organization (WHO) as one of 14 South American countries at risk for YFV transmission based on current or historic reports of yellow fever, plus the presence of competent mosquito vectors and animal reservoirs [11].

Since December 2016, an outbreak of sylvatic YFV is ongoing in Brazil; as at 9 March 2017, there were 371 confirmed and 966 suspected human cases, while a total of 968 epizootics in non-human primates have been reported, of which 386 were confirmed [2]. So far, there has been no evidence for a change from sylvatic to an urban transmission cycle [1]. In addition, Bolivia, Colombia and Peru have reported suspected and confirmed yellow fever cases in 2017 [2].

A subclinical infection with YFV is believed to occur in most infected people. In symptomatic cases, symptoms of general malaise occur after an incubation period of 3–6 days (range 2–9 days), followed by remission of the disease in the majority of patients. However, 15-25% of symptomatic persons develop a complicated course of illness, in which symptoms recur after 24–48 hours, with a reported mortality of 20-60% [7,12]. This phase is characterised by fever, abdominal symptoms, severe hepatic dysfunction and jaundice, multi-organ failure and haemorrhagic diathesis. As no specific antiviral treatment is currently available, treatment consists of supportive care [7,12].

## Discussion

Although Suriname is considered to be endemic for YFV, no human cases have been officially reported since 1971 [13]. With a population of ca 570,000 people, Suriname has a YFV vaccination coverage of 80–85% in infants [14]. Although WHO recommends vaccination for travellers to countries with risk of YFV transmission like Suriname, sporadic cases of imported yellow fever in returning travellers have been reported for example in Europe, the United States and Asia [15-17], with three reported cases related to the ongoing YFV outbreaks in South America in European travellers since 2016 [18,19]. The establishment of ongoing YFV circulation in Suriname extends the current YFV activity in South America to five countries [2]. However, despite the presence of competent *Ae. albopictus* mosquitoes in France [20] and *Ae. aegypti* in Madeira, the risk for YFV transmission in Europe is currently considered to be very low due to the lack of vector activity [18]. An effective, safe live-attenuated YFV vaccine is available for people aged ≥ 9 months and offers lifelong immunity [7]. Vaccination is advised by the WHO for all travellers to Suriname, for the coastal area as well as the inlands [21]. With regard to yellow fever, pre-travel health advice should take into account destination, duration of travel, season and the likelihood of exposure to mosquitoes (in rural areas, forests versus urban areas), and potential contraindications for vaccination with a live-attenuated vaccine.

The multi-country YFV activity might reflect current, wide-spread ecological conditions that favour elevated YFV transmissibility among wildlife and spill-over to humans. Thorough sequence analysis of currently circulating strains in Brazil, Bolivia, Colombia, Peru and Suriname should provide insight whether the human cases in these countries are epidemiologically linked or represent multiple, independent spill-over events without extensive ongoing community transmission. Because of its potential public health impact, our case of yellow fever was notified to the WHO and the European Union Early Warning and Response System on 9 March 2017, according to the international health regulations [22].

## Conclusion

Clinicians in non-endemic countries should be aware of yellow fever in travellers presenting with fever, jaundice and/or haemorrhage returning from South America including Suriname. This case report illustrates the importance of maintaining awareness of the need for YFV vaccination, even for countries with risk of YFV transmission that have not reported cases for decades.
